# Box-Behnken design (BBD) for optimization and simulation of biolubricant production from biomass using aspen plus with techno-economic analysis

**DOI:** 10.1038/s41598-024-71266-w

**Published:** 2024-09-18

**Authors:** Eman M. Abdel Hamid, Amer M. Amer, Ahmed K. Mahmoud, Eslam M. Mokbl, Mazen A. Hassan, Mohamed O. Abdel-Monaim, Rana H. Amin, Kareem M. Tharwat

**Affiliations:** Chemical Engineering Department, Egyptian Academy for Engineering and Advanced Technology (EAEAT), Km 3 Cairo-Belbeis, Desert Road, PO box 3056, Cairo, Arab Republic of Egypt

**Keywords:** Biolubricant, Biodiesel, Optimization, RSM, Aspen plus, Animal fats, BBD, Chemical engineering, Chemistry, Biosynthesis

## Abstract

The growing concern and limitations for existing lubricants have driven the need for biolubricants, extensively proposed as the most suitable and sustainable lubricating oils. Biolubricant refers to lubricants that quickly biodegrade and are non-toxic to humans and aquatic habitats. Over the last decade, there has been a significant increase in the production of biolubricants due to the rising demand for replacing petroleum-based lubricants with those derived from renewable sources like vegetable oils and lipase that are used in various applications. In this study biodiesel (FAME) produced from blending animal fats and waste cooking was used as a raw material with ethylene glycol for biolubricant production using a transesterification reaction in the presence of calcium oxide which considers the newest and novel part as there is no production of biolubricant from animal fats and waste cooking oil in previous researches. The reaction parameters of biolubricant production were optimized using response surface methodology (RSM) with the aid of Box Behnken Design (BBD) to study the effect of independent variables on the yield of biolubricant. These variables are temperature ranging from (100–150 °C), reaction time ranging from 1 to 4 h, and FAME (Fatty Acid Methyl Ester) to alcohol molar ratio ranging from (2:1) to (4:1). The highest biolubricant yield is 91.56% at a temperature of 141 °C, a FAME/alcohol molar ratio of 2:1, and 3.3 h. Various analyses were performed on the produced biolubricant at the optimum conditions. The results include a pour point of -9 °C, a flash point of 192 °C, a kinematic viscosity at 40 °C of 10.35 cSt, a viscosity index of 183.6, an ash content of 0.76 wt.%, and a carbon residue of 1.5 wt.%, comparing favorably with the ISO VG 10 standard. The production process of biolubricant was simulated with Aspen Plus version 11 using a Non-Random Two-Liquid (NRTL) fluid package. The simulation results indicated that the production process can be applied on an industrial scale. Economic analysis was performed on the biolubricants production plant. The total capital investment was $12.7 M with a payback period of 1.48 years and an internal rate of return (IRR) of 67.5% indicating the suitability and profitability of the biolubricant production.

## Introduction

Population growth is continuing and could reach 9 billion by the end of 2050^1^. It causes an increase in industrial activities which increases the demand for energy resources, resulting in nonrenewable energy shortages in recent years^2,3^.

Over 40 million metric tons of lubricating oils are generated annually in the world; approximately half of this quantity is lost to the environment^4^. Lubricants are grouped into three categories based on the main constituents and characteristics of the basic oil: mineral (petroleum source), synthetic (such as silicone, polyalkylene glycols, polyalphaolefins, synthetic esters, etc.), and biolubricant/green lubricant^5^. In order to minimize the friction and the released heat produced when surfaces rub against one another, lubricants are generally used extensively in the following industries: mining, forestry, transportation, chainsaws, mining, mining, and machinery^6^. However, the environmental use of lubricants is greatly threatened by spillage and evaporation, which can seriously impact aquatic life, soil, drinkable water supplies, plants, and people^7^. For instance, one kilogram of mineral oil can contaminate one million liters of water, leading to long-term respiratory issues, inflammation, and cancerous effects. Thus, it has been determined that one of the biggest risks to human welfare is lubricant contamination^8,9^. The sustainability development strategy that agreed with Egypt Vision 2030 has identified pollution reduction and integrated waste management as strategic goals to reduce air pollution and pollution from untreated waste, both of which have serious environmental and health consequences^10^. Scientists are encouraged to develop sustainable and renewable energy in response to global warming concerns. Scientists are continually on the lookout for alternatives to lubricating oil such as biolubricant, which are regarded as efficient renewable sources. Because it is environmentally friendly, renewable, and highly biodegradable, it is an important strategy for climate change mitigation and adaptation. There are some vegetable oils and animal fats available, such as jatropha curcas, coconut, sunflower, peanut, palm oils, rendered fats, and fish oils that can be used as raw material for biodiesel production^11–16^.

The use of bio-based lubricants, including bio-based greases, significantly improves lubricant biodegradability, resulting in less air, soil, and water pollution and fewer health risks^17^. Biolubricants are important for many applications because they are biodegradable, have low ecotoxicity^18–20^, and don't contribute to the emissions of volatile organic compounds. However, their low-thermal oxidation stability and low-temperature characteristics issues make their widespread and commercial usage in industries difficult, necessitating the usage of additives^21,22^. Therefore, biolubricants are lubricants derived from renewable natural raw materials, such as vegetable and animal oils, that are safe for use around people and other living things^23^.

Biolubricants offer significant advantages as replacement lubricants for industrial and maintenance applications due to their superior intrinsic qualities. Because of their advantages for the environment, biolubricants can be utilized in delicate situations and reduce pollution. Biolubricants can be employed in industrial oils such as machine oil, hydraulic oil, and automotive oil^20^. Transesterification, estoile production, epoxidation with the subsequent ring-opening, and acetylation reaction are a few examples of traditional chemical modification techniques^24,25^. It is also critical to choose the best modification route for their synthesis because vegetable oils have some drawbacks, such as oxidative and hydrolytic instability, lubricating limitations at low temperatures, and low compatibility with paints and sealants^26^. Animal fats have great potential in the biofuel industry in the twenty-first century biofuel industry due to increasing energy costs^27^, The European Union alone produces around 17 million tons of waste annually^28^. The usage of nonedible vegetable oils is preferred as a feedstock of biolubricant production that doesn’t affect food security. Many researchers have investigated various non-edible oils such as jatropha oil, neem oil, rubber seed oil, waste cooking oil, cotton seed oil, tilapia oil, palm kernel oil, canola oil, and soybean oil^14,21,29–40^. Due to its renewability and sustainability, the transesterification method is the common process used for biolubricants production. The conversion of biolubricant is affected by the catalyst type, the ratio between glycerides to alcohols, the content of fatty acid in the feedstock, and the temperature^22^.

Biolubricants are specially designed to biodegrade with the assistance of biological microorganisms. They boast high cloud and flash points, as well as high pour points, leading to solidification at low temperatures. Moreover, they offer low friction and wear properties, but do suffer from low oxidation stability at high temperatures, which can hinder lubricating oil performance and lead to increased polymerization and acidity^26^.

Kamyab et al.^35^ investigated the impact of time, temperature, and canola oil ethyl ester (FAEE) on the trimethyl propane (TMP) molar ratio, along with the catalyst concentration of potassium carbonate on the biolubricant yield, they discovered that the highest yield, reaching 85%, is achieved at a temperature of 130 °C, a reaction time of 3 h, an FAEE to TMP molar ratio of 3.1:1, and a catalyst loading of 1% of potassium carbonate. Also, Hamnas and Panicker^41^ discussed the feasibility of blending mustard oil and castor oil in the synthesis of biolubricant as an alternative to commercial engines. Shrivastava et al.^36^ used soybean oil as a feedstock for the synthesis of biolubricant in the presence of Zn Al hydrotalcite as a catalyst. Prasannakumar et al.^42^ studied the synthesis of biolubricant from cashew nut shell oil as a non-edible oil. Shrivastava et al.^43^ studied the usage of Ni–Al Hydrotalcite as a heterogeneous catalyst in the synthesis of biolubricant while Sharma et al.^44^ investigated the behavior of the viscous flow of biolubricant produced from karanja oil.

Due to the great concern for solving climate change problems, there is a continuous search to find alternatives to fossil fuels. This is achieved by replacing petroleum-dependent products with renewable energy-dependent products, which represents a central strategy to adapt to climate changes and be eco-friendly, renewable, and more biodegradable.

In order to generate a high yield of biolubricant, a unique synthetic strategy for the transesterification reaction was described in the current research. Reaction temperature, time, and molar ratio are the main reaction variables that affect the yield of biolubricant. Response surface methodology has been used to assess the impact of these variables and their interactions on the biolubricant yield. A group of statistical and mathematical methods known as response surface methodology are applied to the modeling and analysis of situations where multiple variables affect an interest response. The goal is to maximize the output variable, or response, which is affected by multiple independent variables, often known as input variables or factors^45–47^. The variables in each experiment are various operating condition values or levels. Some might be quantitative (feed rates, temperatures, time, etc.), while others might be categorical (raw material source, for example). In actuality, categorical variables need to be managed independently by contrasting our optimal operational settings about the quantitative variables in various combinations of the categorical ones. Fitting first-order (linear) or second-order (quadratic) functions of the predictors to one or more response variables is the primary method for quantitative variables. Based on the features of the fitted surface, one can determine the proper course of action^48^.

The aim of this research is the production of biolubricant by blending animal fats and waste cooking oil using a double transesterification method in the presence of a heterogeneous catalyst and finding the optimum condition to produce the highest yield of biolubricant which is not discussed in the previous studies. With the aid of the Aspen Plus V.11, the production process of biolubricants was simulated. The purpose of the simulation was to perform mass and heat balance as well as investigate the industrial applicability of the process under examination. An economic study is performed on the production process to determine the feasibility and profitability of the project industry.

## Materials and methodology

**Fig. 1:**
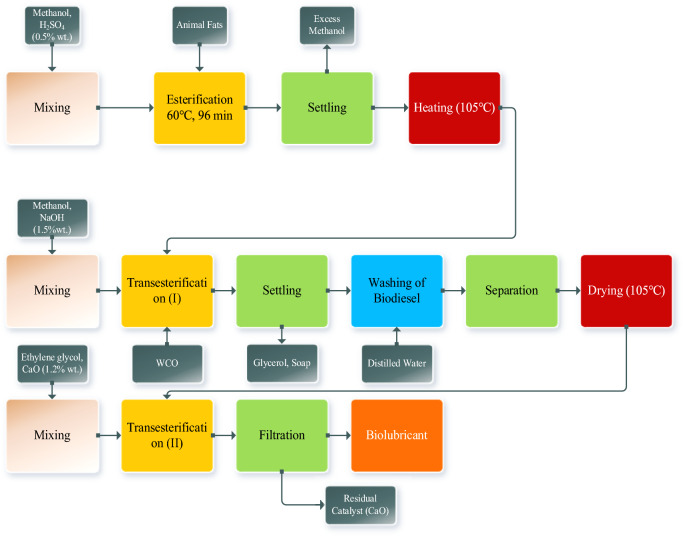
Experimental work procedure.

This work aims to produce an ecofriendly biolubricant from biomass which is a blending of waste cooking oil and animal fats that yields a high lubricity of the biolubricant by passing through the esterification of the raw material to be treated before biodiesel production by transesterification reaction and finally goes to the production of biolubricant by a second transesterification reaction. Each step will be discussed in the following sections and also Fig. 1 illustrates the full experimental procedure for biolubricant production.

### Raw materials

The raw materials used in the production of biolubricant are animal fats from the slaughterhouse and waste cooking oil (WCO) collected from home and some local restaurants. Concentrated sulfuric acid (98%) was purchased from El-Gomhouria Company for Chemicals and Medical Appliances, Egypt. Methanol (99.9%) and pellets of sodium hydroxide (98%) were from El Shark El-Awsat Company for Chemicals, Egypt. Ethylene glycol (99%) and calcium oxide (95%) were purchased from Alpha Chemical Company, Egypt.

### Pretreatment of animal fats

200 g of animal fats was washed and cut into small pieces then heated at a temperature of 400 °C for 10 min^49^ to separate the suspended solids and evaporate the moisture content^50^. The hot mixture was filtrated using a cotton sieve to obtain oil. The treated animal fats were stored for the esterification reaction.

### Esterification of animal fats

Esterification was performed on the animal fat waste that contained high-free fatty acids according to Eq. (1). Esterification with acid catalysts was recommended to reduce FFAs to less than 2% using base catalyst produces soap which reduces the yield of biodiesel and slows down the reaction rate^51^.

1 $$FFA+MeOH\leftrightarrow FAME+Water$$

The treated animal fats were heated at a temperature of 60 °C, and a mixture of methanol (9:1 methanol to oil molar ratio) and 0.5% wt. of conc H_2_SO_4_ were added to the animal fats for 96 min^49^. Then the mixture was left to settle overnight to produce two different liquid phases. The upper layer contains the unreacted H_2_SO_4_ catalyst, methanol, and impurities, while the bottom layer contains the treated animal fats. Then the treated fats were heated to 105 °C to remove any traces of methanol and water to be used in the transesterification reaction^52,53^.

### Characteristics of raw materials

Animal fats and vegetable oils have identical requirements for petroleum and non-petroleum oils. Vegetable oils and animal fats share common physical properties and produce similar environmental effects. Density, free fatty acids (%FFA), and water content were determined.

The free fatty acids (%FFA) were estimated according to the method of the American Oil Chemist’s Society. In the present work, 0.5 g of the oil was weighed in a flask of 250 ml and then diluted with 50 ml of isopropyl alcohol followed by the addition of 3 drops of 0.1% phenolphthalein solution. The mixture was shaken till the dissolved oil then the solution was titrated with a solution of potassium hydroxide with a concentration of 0.1 N till the formation of pink color lasted for 30 s^49^. Equation (2) was used to determine the FFA.

2 $$\text{\%}FFA=\frac{{V}_{KOH}*Normality \left(KOH\right)*{M}_{KOH}}{2*W}$$

where V_KOH_ is the volume of potassium hydroxide used in the titration, MKOH is the molecular weight of KOH, and W is the weight of oil.

### Experimental procedure

#### Synthesis of biodiesel

Biodiesel is produced using a transesterification reaction in the presence of a base catalyst, in which triglycerides react with alcohol. As illustrated in Eq. (3), the hydroxyl groups of the alcohol replace the triglyceride fatty acids to form fatty acids methyl esters and glycerol as a byproduct. After glycerol separation, the main product is fatty acids methyl ester (biodiesel). The biodiesel mix is substantially less viscous without the glycerol backbone than the original vegetable oil or animal fat, and its fuel qualities are ideal for powering diesel engines^54^.3

A blend of waste cooking oil and animal fats was used to produce the biodiesel by transesterification reaction, waste cooking oil was added with different percentages ranging from 0 to 100% by increasing 20%. The blended mixture was heated to 60ºC and then mixed with a mixture of sodium hydroxide and methanol which was placed in a water bath and the flask was well equipped with a reflux condenser to avoid methanol loss. The transesterification reaction conditions are at a temperature of 60 °C, methanol to oil molar ratio (9:1), a reaction time of 90 min, and (NaOH) catalyst dosage (1.5% wt.) determined according to Eq. (4)^55^. At the end of the transesterification reaction, the mixture is left to settle overnight in a separating funnel. Biodiesel is separated and then washed using warm distilled water at a temperature of 80 °C. The washed biodiesel was heated at 105 °C for 30 min to remove the moisture content. The same procedures will occur with different percentages of waste cooking oil and treated animal fats blending. After choosing the suitable ratio of both fats and WCO, then varying the methanol to oil molar ratio from (12:1 to 6:1) and time-varying from (90–180 min).

4 $$NaOH\left(\%\right)=\left(FFA\right)\times (0.144) + 1\%$$

#### Characteristics of biodiesel

The main characteristics of the biodiesel produced were determined at the optimum conditions such as Fourier Transform Infrared Spectroscopy (FTIR), density using a hydrometer, calorific value, kinematic viscosity, acid value, cetane index, and sulfur content. The optimum samples were studied, and compared with the limits established in the standard. FTIR allows access to many of the important functional groups that are connected to biodiesel content, the creation, and breakdown of ester linkages, synthesis, and degradation of OH. The FTIR model used is Class 1 Laser Product IEC/EN 60825-1/A2:2001 Avatar Series (USA) in The Egyptian Academy for Engineering and Advanced Technology. Also, the hydrocarbon composition was determined using gas chromatography, Helium was utilized as the carrier gas at a flow rate of 1.5 mL/min while the auto-injector on the GC–MS Shimadzu GC 17-A gas chromatograph apparatus was run in the splitless mode at 220 °C. After injecting 1 L of the sample, the oven's temperature was raised from 80 to 200 °C at a rate of 10 °C/min. The temperature was raised to 270 °C at a rate of 8 °C/min, held there for 1 min, and then the biodiesel test was conducted to verify the quantity of methyl ester present.

#### Synthesis of biolubricant

Biolubricant was produced by a transesterification for the produced biodiesel from the waste cooking oil and animal fats blending using a polyol as ethylene glycol (EG) using calcium oxide as a heterogeneous catalyst, where methanol in this reaction is a byproduct as shown in Eq. (5). The following steps explain the production of biolubricant as shown in Fig. 1^38^.

**Fig. 2:**
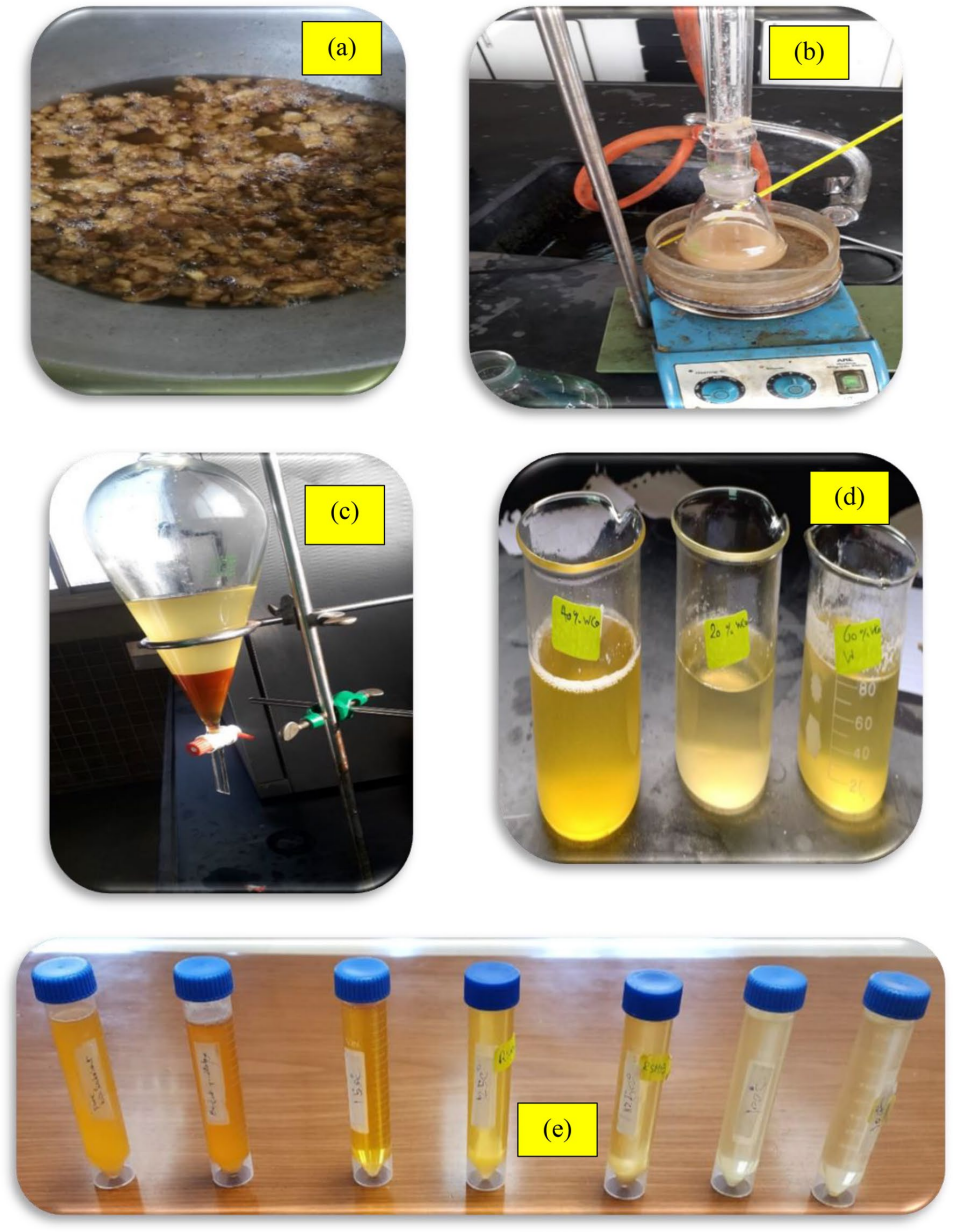
Preparation of biolubricant; (**a**) Melting of animal fats, (**b**) Transesterification (I), (**c**) Separation of biodiesel and glycerol, (**d**) Biodiesel from different percentages of blending animal fats and WCO, (**e**) Different runs of biolubricant synthesis.

A hot bath was used to heat a combination of biodiesel (FAME) and ethylene glycol using oil as a heating media at temperatures ranging from 100 to 150 °C. The thermometer was placed in the opposite neck of a two-neck glass flask linked to the water condenser. The water condenser was tilted and linked to a methanol collecting flask to avoid a reversible reaction. At the end of the reaction, the calcium oxide catalyst was filtered from the reaction mixture. Reaction conditions ranged from 2:1 to 4:1 for FAME to EG molar ratio, CaO catalyst 1.2% (wt/wt) with a reaction time ranging from 1 to 4 h. The produced biolubricant was filtrated to eliminate the catalyst. The real photos from the experiments are displayed in Fig. 2.

A reusability test was performed to determine the catalyst efficiency. The highest yield of biolubricant synthesis at the optimum condition that obtained from the Design Expert software. After performing the first reaction the catalyst was then separated from the biolubricant and then washed with methanol followed by drying at a temperature of 105 °C in an oven till the weight became constant to be used again in biolubricant synthesis at the same conditions. The previous procedures were repeated several times.5

#### Characteristics of biolubricant

The characteristics of the produced biolubricant at the optimum conditions were determined and compared with the different ISO viscosity grades. The lowest temperature at which a lubricant loses its capacity to flow is known as the pour point. It is directly correlated with the viscosity index in biolubricants. The pour point amount also depends on the raw material of the biodiesel. It can be measured using ASTM D5949 with the aid of Peltier device at a rate of 1.5 ± 0.1 °C/min. The kinematic viscosity has been measured at 40 and 100 °C using SVM 3001 Stabinger viscometer Anton Paar.

The ASTM D93 technique was used with a Pensky-Martens closed-cup tester to determine the flash point of the biodiesel. The automated Pensky-Martens closed-cup equipment was used to measure the flash points of biodiesel at a temperature ranging from 60 to 190 °C. Carbon residue was measured using furnace met while ash content was investigated using PYRO Microwave Ash Furnace Meslo.

### Experimental design using response surface methodology

Design-Expert software version 13 was used to optimize the independent variables of the reaction. In the current study, the experimental design used was Box Behnken Design (BBD) with three variables resulting in 17 experiments. Response surface methodology (RSM) was implemented to optimize the biolubricant production by changing the main factors affecting the yield of biolubricant which are FAME to alcohol molar ratio (2–4), reaction temperature (100–150 °C) and reaction time (1–4 h). Table 1 illustrates the three main variables that were used in the design of the experiments which were performed in random arrangement to avoid errors. With the aid of the regression analysis, the response function was determined according to the second-order polynomial^56^ as shown in Eq. (6).

6 $$\text{Y }=\upbeta 0 +{\sum }_{i=1}^{k}{B}_{i}{X}_{i}+{{\sum }_{i=1}^{k}{B}_{ii}X}_{i}^{2} +{\sum }_{i=1, i<j}^{k-1}{\sum }_{i=2}^{k}{B}_{ij}{X}_{i}{X}_{j}$$

where Y represents the predicted response yield for biolubricant and the regression coefficients were represented as βi, βii, and βij. The design of the experiment is illustrated in Table 2.

**Table 1:** Limits of independent variables used in the design of experiments.

Process variables	Unit	Variables levels		
		− 1	0	1
Time	h	1	2.5	4
Temperature	°C	100	125	150
FAME (80% WCO and 0% animal fats)/alcohol molar ratio	–	2	3	4

**Table 2:** Actual values of design of experiments.

Run	Temperature (°C)	Time (h)	FAME/alcohol (mol/mol)
1	125	2.5	3
2	125	1	4
3	125	4	2
4	150	1	3
5	150	2.5	2
6	150	2.5	4
7	125	2.5	3
8	150	4	3
9	100	2.5	2
10	100	4	3
11	125	2.5	3
12	100	2.5	4
13	125	1	2
14	125	2.5	3
15	125	4	4
16	125	2.5	3
17	100	1	3

The yield of the biolubricant can be determined using Eq. (7)^38^.

7 $$Yield, (\%)=\frac{Mixture\ weight\ before\ reaction-Mixture\ weight\ after\ reaction}{Theoritical\ mass\ of\ Methanol\ produced\ according\ to\ the\ limiting\ reactant}\times 100$$

### Modeling and simulation of biolubricant production

The production of biolubricant was simulated at the optimum condition using Aspen Plus version 11. The Aspen Plus V.11 is a software program used for process simulation including the design of chemical plants and dynamic simulation. A mathematical model can be established affording to the actual production process and gaining the anticipated results by varying the conditions of the reaction. Due to the existence of polar components in the process, the thermodynamic fluid package that might be employed for this process is Non-Random Two-Liquid (NRTL) activity coefficient model^57^.

The chemical components include triglycerides (triolein), methanol, ethylene glycol, sodium hydroxide, glycerol, oleic acid (fatty acid), fatty acid methyl esters (methyl oleate, C_19_H_36_O_2_), calcium oxide, water, soap (sodium oleate), sodium sulfate and ethylene glycol di-ester (biolubricant). These components were used in the library components of Aspen Plus. In the present study, WCO was introduced as a triolein (C_57_H_104_O_6_) component in Aspen Plus. The animal fats were assumed to be a combination of 7% wt. of oleic acid and 93% wt. of triolein.

**Fig. 3:**
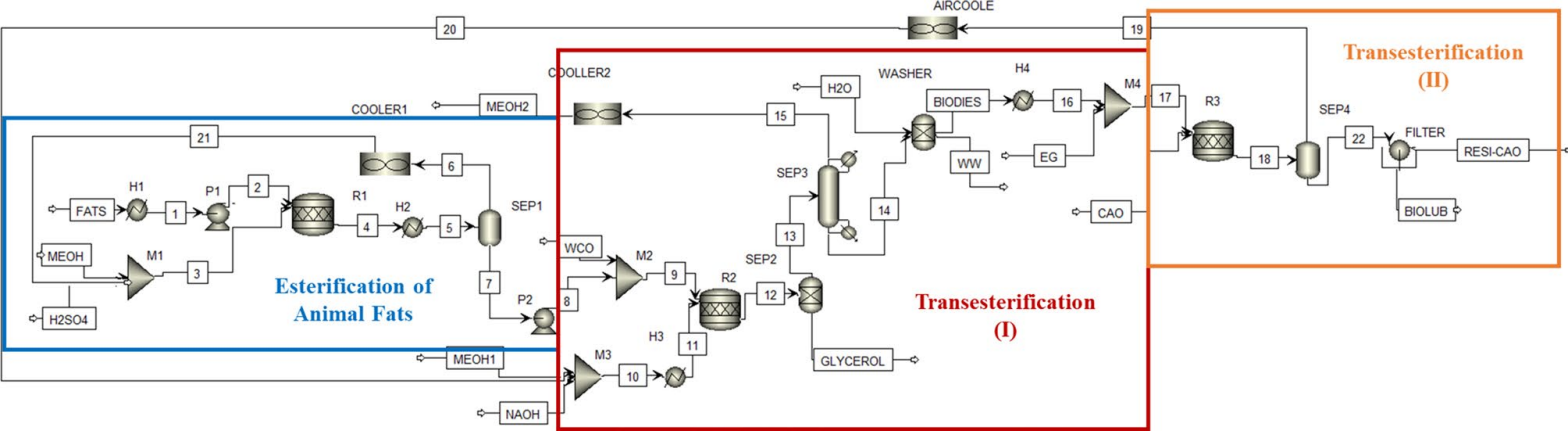
Simulation of biolubricant production using aspen plus.

The production of biolubricant from the blending of waste cooking oil (WCO) and animal fats was simulated through three main parts as shown in Fig. 3. The first part is the esterification of animal fats as they react with the mixture of methanol and H_2_SO_4_ as a catalyst to decrease the free fatty acids of animal fats (treated animal fats). The second part is the transesterification of blending both WCO and treated animal fats that reacted with a mixture of methanol and NaOH as a homogenous catalyst to produce biodiesel. The third part is second transesterification which is performed after reacting the produced biodiesel with the ethylene glycol to produce biolubricant.

Animal fats with a mass flowrate 500 kg/h were heated to a temperature of 60 °C using the heater (H1) then pumped in pump (P1) to 101.3 kPa and mixed with a mixture of sulfuric acid and methanol in a mixer (M1). The mixed stream is sent to a conversion reactor (R1) to produce animal fats with low fatty acids according to Eq. (1). The animal fats with low fatty acids were heated to a temperature of 105 °C in a heater (H2) and then entered into a flash separator (SEP1) to separate the unreacted methanol vapor and water traces from the treated animal fats. The unreacted methanol was cooled in a cooler (COOLER1) to a temperature of 60 °C and recycled back to the mixer (M1). The treated animal fats were pumped in a pump (P2) and then mixed with 2000 kg/h of WCO to be able to enter a second conversion reactor (R2) along with a mixture of sodium hydroxide (1.5%) and methanol with a molar ratio (6:1) methanol to oil at a temperature of 60 °C. In this reactor, several reactions take place which were transesterification, saponification due to the reaction of fatty acid (oleic acid) and sodium hydroxide, and finally neutralization due to the reaction of residual sulfuric acid present in treated animal fats with sodium hydroxide. The product was sent to a liquid–liquid gravity separator (SEP2) to separate the light liquid (methanol and biodiesel) as the top product and the heavy liquid (glycerol and sodium sulfate) as the bottom product. The light liquid stream was sent to a flash separator to separate excess methanol as a top product which was cooled in a cooler (COOLER2) to 30 °C to send to a storage tank. The bottom product (biodiesel) was sent to a washer tank to remove the residual soap and then heated to a temperature of 141 °C. The heated biodiesel was sent to a third reactor (R3) along with ethylene glycol at a temperature of 141 °C in the presence of calcium oxide as a heterogeneous catalyst according to the optimum conditions.

The byproduct methanol was obtained after introducing the product stream into a flash separator (SEP4) to produce pure methanol as a top product and biolubricant as a bottom product. The residual calcium oxide was removed from the biolubricant using a filter. The produced methanol from (SEP4) was cooled to a temperature of 60 °C to recycle back to a mixer (M3).

### Economic analysis

Besides the technical assessment, economic analysis, environmental impact, and social factors are considered the main aspects of any project assessment. Economic analysis is used to determine the feasibility and profitability of the project industry. The overall economic performance of the biolubricants production plant such as fixed capital cost, manufacturing cost, and profit can be estimated when identifying the cost of raw materials, process technology, and plant capacity.

The total capital investment is the summation of working capital investment and fixed capital investment that includes the cost of equipment, installation, design, and construction^58^.

Net present value (NPV) is the difference between the present cash inflows and outflows that represent the economic statistics. NPV can be calculated using Eq. (8)^59^.

8 $$NPV=\sum_{n}\frac{{C}_{t}}{{(1+r)}^{n}}-{C}_{o}$$

where C_t_ is the net cash inflow, C_o_ is the initial investment cost, n is the project period time and r is the discount rate.

Manufacturing cost is the summation of fixed and variable costs. Fixed costs include labor, insurance, depreciation, plant overhead, rent, interest, and royalties while the variable cost is the summation of raw materials and utilities. It was assumed that the labor cost for the factory is USD 76,595 with two shifts and operating working hours of 8 per shift. The cost of supervisors is about 20% of the labor cost.

In the current economic study, the following assumptions are considered:

- The operating hours per day are 16 h, and 300 working days
- All costs in the current study are in US dollars. The chemical engineering plant cost index of $800 is used to update the cost of the equipment.
- The lifetime of the project is 25 years
- The Aspen Process Economic Analyzer is used to determine some equipment and utilities costs including (electricity, steam, water, and hot oil)

The profitability of the project is the internal rate of return (IRR), the project can be considered highly profitable when the value of IRR is high.

## Results and discussions

### Characteristics of raw materials

The characterization of WCO and fats was carried out in the laboratory to determine the density (ρ), moisture content, and FFA% as shown in the following sections.

The free fatty acids of animal fats were measured before the esterification process, which equals 7%, and this value is very high, due to the presence of some saturated fatty acids in a large proportion of the fat, such as palmitic acid (21.6 wt%), stearic acid (17.7 wt.%), as well as some unsaturated acids such as oleic acid (31.5 wt.%), and after applying the esterification process, the presence of these acids was reduced to be 4%^28^.

The free fatty acids of the WCO were measured and it was equal to 1%, and this percentage is acceptable. The reason for this result is the presence of only some unsaturated acids, namely oleic acid (45.15 wt.%) and linoleic acid (39.74 wt.%)^60^.

The density of WCO and animal fats were measured and it was found to be 890 kg m^−3^ and 770 kg m^−3^ respectively. The water content of WCO and animal fats was found to be 0.04% and 0.085% respectively. The low water content in animal fats is due to the evaporation of all moisture in the pretreatment of animal fats. When the percentage of water content is low that means low saponification occurs.

### Results of biodiesel production

#### Effect of different parameters on biodiesel yield

##### Effect of blending animal fats and WCO on biodiesel yield

**Fig. 4:**
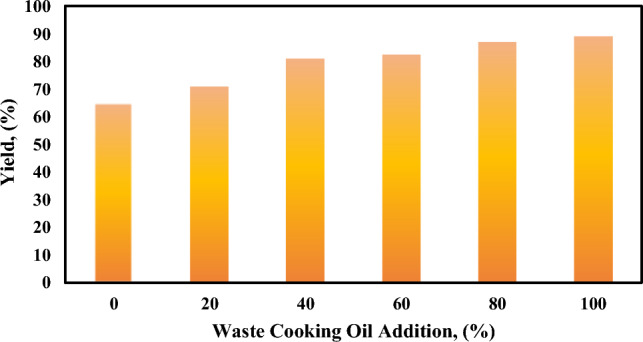
Effect of blending animal fats and WCO.

Increasing the addition of WCO to the animal fats causes increasing in biodiesel yield as shown in Fig. 4. This returns to the high free fatty acid content in the animal fats because the total FFA content reacts with the NaOH catalyst to form soaps as shown in Eq. (9). This saponification reaction is undesirable because it lowers the yield of the produced biodiesel and makes the separation of glycerol difficult. The yield at 80–100% WCO addition is almost constant because the FFA content in the 80% WCO blend is around the same as that in the 100% WCO blend^61^. The calorific value for 80% WCO was found to be 42.5 MJ kg^−1^ which is higher than biodiesel from 100% WCO 39.4 MJ kg^−1^ at the same operating conditions. The blend with 80% WCO and 20% animal fats was chosen as the optimum blending for biodiesel production.

9 $${R}_{1}COOH+NaOH\to {R}_{1}COONa+ {H}_{2}O$$

##### Effect of alcohol to oil molar ratio on biodiesel yield

**Fig. 5:**
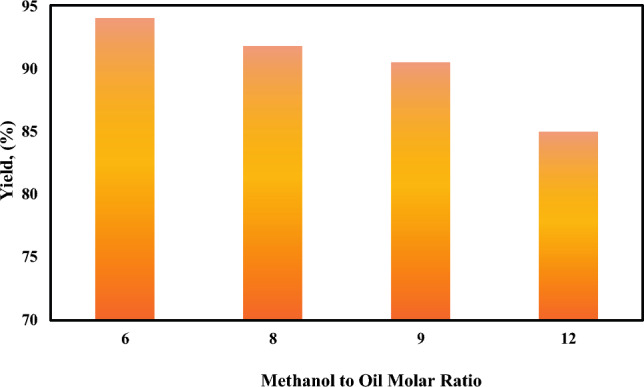
Effect of methanol to oil molar ratios.

The methanol-to-oil molar ratio is considered to be one of the most important factors affecting the yield of biodiesel due to the reversibility of the reaction, increasing the methanol-to-oil molar ratio causes the reaction to shift in the right direction. The different methanol-to-oil molar ratio was studied for biodiesel production at 20% animal fats and 80% WCO. The result indicated that increasing the ratio of methanol to oil decreases the biodiesel yield as shown in Fig. 5. Increasing the molar ratio increases the glycerol production and deactivates the catalyst which hinders the reaction resulting in lower biodiesel yield and reducing its effectiveness.

Excess methanol blurs the separation border between produced glycerol and biodiesel which makes the extraction of the biodiesel more difficult, while the presence of methanol in the final product is undesirable because it reduces the flashpoint of the biodiesel. Based on this finding, a molar ratio of 6:1 was determined to be the optimum condition^21,22^.

##### Effect of reaction time on biodiesel yield

**Fig. 6:**
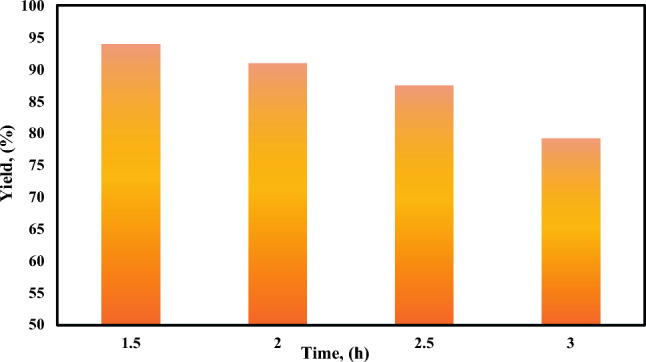
Effect of reaction time.

The effect of reaction time on the biodiesel yield at a temperature of 60 °C was investigated by varying time from 1.5 to 3 h. It was observed that the yield decreases with increasing reaction time as shown in Fig. 6. This occurs due to the evaporation of methanol which has a low boiling point at high reaction time. This is also related to the molecular structure of saturated fatty acids that exist in animal fats. These fatty acids have a greater activation energy and hence require more heating time to react. As a result, the biodiesel yield increased significantly at 1.5 h, reaching a maximum value as the activation energy was reached and the process reached equilibrium, after which the yield decreased due to the reversible nature of the transesterification reaction. The prolonged heating could cause the produced biodiesel to revert to its original state and form more soap, reducing the overall biodiesel yield^62^.

#### Characteristics of biodiesel

##### Infrared spectroscopy analysis (FTIR)

**Fig. 7:**
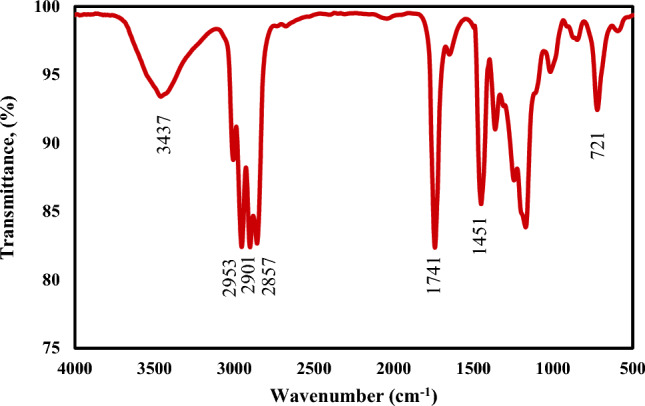
Fourier transform infrared spectroscopy (FTIR) Analysis of the produced biodiesel.

The biodiesel was confirmed by FTIR examination to indicate the different functional groups of the sample due to spectra absorbance as shown in Fig. 7. The absorption bands observed of hydroxy (OH), alkenes (=CH), and a carboxyl group (C=O) at 3437, 2953, and 1741 cm^−1^ respectively. The ester group (C–O) was found at 1171 cm^−1^ and the absorption wavelength of 721 represents alkenes (C=C)^63–65^.

##### Properties of biodiesel

Gas chromatography (GC) is used to identify and quantify individual components of a mixture of volatile compounds. It is used to detect the conversion of triglycerides into diglycerides and then monoglycerides which are broken down further to produce FAME^66^. The amount of saturated fatty acid methyl ester is about 32.6% while the unsaturated fatty acid methyl ester is about 67.4%. Table 3 shows the gas chromatography composition of the produced fatty acids methyl esters (FAME) after transesterification of the triglycerides, which represent a good alternative to diesel fuel^14,65^.

**Table 3:** Gas chromatography composition analysis of the produced biodiesel.

Fatty acids	Composition (wt.%)
Palmitic acid methyl ester (C16:0)	23.93
Stearic acid methyl ester (C18:0)	8.65
Oleic acid methyl ester (C18:1)	25.62
Linoleic acid methyl ester (C18:2)	34.61
Linolenic acid methyl ester(C18:3)	7.18

The comparison between produced biodiesel and their standard specification is illustrated in Table 4. The results indicated that the properties of the produced biodiesel are compatible with the standard specifications.

**Table 4:** Comparing the produced biodiesel with previous studies.

Parameters	Present study	WCO^67^	Beef Tallow^49^	Standard^68^	Method
Density at 15°	873	875	–	860–900 kg/m^3^	EN ISO 3675
Kinematic viscosity at 40°	4.99	2.323	5.4	1.9–6 mm^2^/s	ASTM D-445
Flash point	186	123	154	93 °C	ASTM D-92
Cetane Index	61.64	–	56	≥ 47	ASTM D-976
Acid value	0.33	0.32	0.836	≤ 0.5 mg KOH/g	ASTM D664
Sulfur content	0	–	–	10 mg/kg	ASTM D-4294
Calorific value	42.5	32.3	–	≥ 37.2 MJ kg^−1^	

The results also showed that the sulfur content is zero due to the neutralization reaction that occurs between the residual sulfuric acid present in the animal fats and sodium hydroxide as a catalyst in the transesterification reaction of biodiesel according to Eq. (6).

### Results of biolubricant production

#### Statistical analysis of biolubricant production

##### ANOVA for the production of biolubricant

Table 5 shows the actual and the predicted values of biolubricants yield according to the experimental design program software. RSM was used to develop a mathematical statistics relationship between the independent variables such as reaction time, temperature, and FAME to alcohol molar ratio and the yield of biolubricants. Analysis of variance (ANOVA), analysis of the regression (R^2^), and 3D plotting of the response variables were used to optimize to obtain parameters for the maximum biolubricants production. The results indicated that the quadratic regression model was the best-fitted model to optimize the biolubricant production. Equation (10) illustrates the quadratic model of the yield of biolubricants.

**Table 5:** Box-Behnken design (BBD) experimental results of biolubricant production.

Run	A: Temperature (°C)	B: Time (h)	C: Fame/alcohol	Actual yield (%)	Predicted yield (%)
1	125	2.5	3	82.4	81.68
2	125	1	4	61	61.56
3	125	4	2	85	84.44
4	150	1	3	62.5	63.00
5	150	2.5	2	87	86.44
6	150	2.5	4	88	86.94
7	125	2.5	3	81.7	81.68
8	150	4	3	89	90.12
9	100	2.5	2	44	45.06
10	100	4	3	66.5	66.00
11	125	2.5	3	82.9	81.68
12	100	2.5	4	66	66.56
13	125	1	2	39	39.06
14	125	2.5	3	81.4	81.68
15	125	4	4	84	83.94
16	125	2.5	3	80	81.68
17	100	1	3	26.5	25.37

10 $$Yield = 81.68+ 15.44 A + 16.94 B + 5.5 C - 3.38 AB - 5.25 AC - 5.75 BC - 8.28 {A}^{2}- 12.281 {B}^{2}- 2.15 {C}^{2}$$

ANOVA was used to validate the strength and significance of the results of the experiments and also the effect of these variables and their effect on the response. The ANOVA results are shown in Table 6. The F-value of the regression model is 389.79 and the p-value is almost zero which indicates the significance of the model. The probability of the pure error was represented by (p-value) which was used to check the performance of each regression coefficient. The p-value of the lack of fit is 0.2895 which indicates that the model is sufficient for describing the relation between the independent variables and the response. The p-values of A, B, C, AB, AC, BC, A^2^, B^2^, and C^2^ are all less than 0.05, indicating that the model terms significantly affect the yield of biolubricants.

**Table 6:** Analysis of variance (ANOVA) of the quadratic model of biolubricant production.

Source	Sum of squares	df	Mean Square	F-value	p-value	
Model	5748.50	9	638.72	389.79	< 0.0001	significant
A-Temperature	1906.53	1	1906.53	1163.48	< 0.0001	
B-Time	2295.03	1	2295.03	1400.57	< 0.0001	
C-FAME/alcohol	242.00	1	242.00	147.68	< 0.0001	
AB	45.56	1	45.56	27.81	0.0012	
AC	110.25	1	110.25	67.28	< 0.0001	
BC	132.25	1	132.25	80.71	< 0.0001	
A^2^	288.49	1	288.49	176.06	< 0.0001	
B^2^	634.68	1	634.68	387.32	< 0.0001	
C^2^	19.51	1	19.51	11.91	0.0107	
Residual	11.47	7	1.64			
Lack of fit	6.56	3	2.19	1.78	0.2895	Not significant
Pure error	4.91	4	1.23			
Cor total	5759.97	16				

The competence of development of the model was evaluated by determining the coefficient of variation (CV%), coefficient of determination (R^2^), adjusted, and predicted R^2^ as shown in Table 7. The values of R^2^ and R^2^ adjusted for biolubricant yield were 0.998 and 0.995 respectively. The value of CV% is 1.8 and the standard deviation is 1.28, indicating a small variation from the mean. When the values of R^2^ are near unity and the standard deviation has a small value indicates that the model is a good predicted response^69^.

**Table 7:** Analysis of variance for a quadratic response surface model.

Std. Dev	1.28	R^2^	0.9980
Mean	70.99	Adjusted R^2^	0.9954
C.V. %	1.8	Predicted R^2^	0.9804
		Adeq precision	65.9511

The results also showed that the value of the predicted R^2^ is very close to the adjusted R^2^. Adeq Precision is used to determine the ratio between the signal to noise, it is desired that this ratio is more than 4. It was found that the ratio is 65.95 which is an adequate signal that is used in the navigation of the design space.

**Fig. 8:**
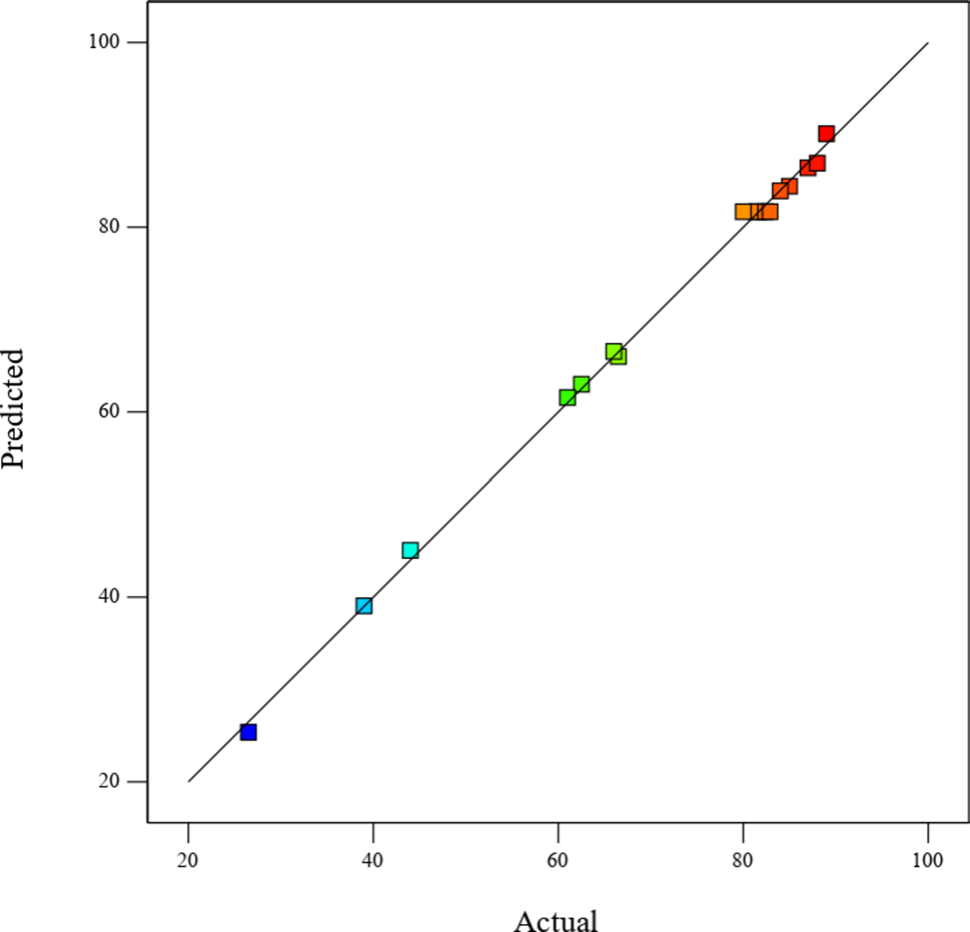
Relation between the predicted and actual experiment values of biolubricant production.

Figure 8 shows a relation between the predicted response obtained from values of the functions produced from the model evaluation and the actual response represented by the experimental design. The figure shows a good agreement and excellent correlations.

##### Interaction of different variables on biolubricant production

**Fig. 9:**
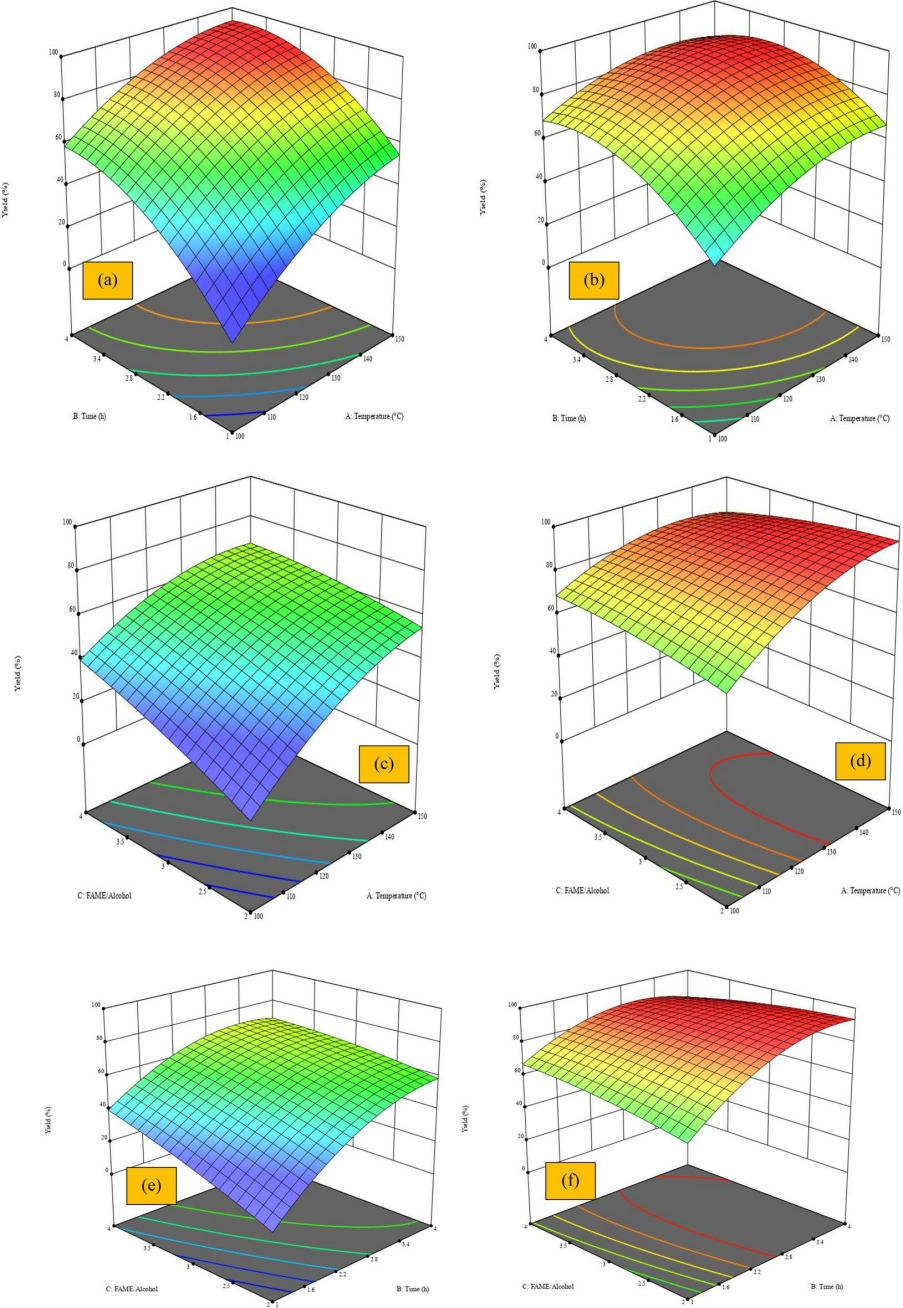
3D Plot of response surface for the effect of different factors on the production of biolubricant, (**a**) Effect of time and temperature at (FAME/Alcohol) molar ratio 2, (**b**) Effect of time and temperature at (FAME/Alcohol) molar ratio 4, (**c**) Effect of temperature and (FAME/Alcohol) molar ratio at 1 h, (**d**) Effect of temperature and (FAME/Alcohol) molar ratio at 4 h, (**e**) Effect of time and (FAME/Alcohol) molar ratio at 100 °C, and (**f**) Effect of time and (FAME/Alcohol) molar ratio at 150 °C

The interaction of time and temperature on the yield of biolubricants at different molar ratios of FAME to alcohol was illustrated in Fig. 9-a,-b varying from 2 to 4. The results indicated that increasing reaction time and temperature causes increasing biolubricant yield from 10% to about 90% at temperatures of 100 and 150 and reaction times 1, and 4 h respectively when using a minimum FAME to alcohol molar ratio (2). The concentration of FAME influenced the kinetics and the equilibrium causing the reaction to shift towards the production of biolubricant^19,69^. More FAME could be used to accelerate the reaction for a higher yield of biolubricant in a shorter time. High molar ratios are desirable for a specific reaction time and temperature since they produce a high biolubricant yield^45^.

Figures 9-c,-d show the interaction of FAME to alcohol molar ratio and temperature on the yield of biolubricant at different reaction times varying from 1 to 4 h. The results indicated that increasing the molar ratio and temperature causes increasing in biolubricant yield from 10 to 65% at the minimum reaction time of 1 h. Increasing time from 1 to 4 h causes a sharp increase in the biolubricant yield and almost remains constant due to reaching the equilibrium. At low temperatures, a longer reaction time doesn't improve the effects of catalytic activity. High temperatures also led to increasing the mass transfer between the reactants and enhanced catalyst particle dispersion in liquid media^70^.

Figures 9-e,-f show the interaction of FAME to alcohol molar ratio and time on the yield of biolubricant at different temperatures ranging from 100 to 150 °C. At lower temperatures, increasing FAME to alcohol to molar ratio and time causes increasing in yield from 10% to about 65%. Increasing temperature causes increasing in biolubricant yield due to the high kinetics rate and collision of molecules which is compatible with the previous studies^69,71^.

##### Optimization of biolubricant production

The software Design Expert optimized 100 solutions for the synthesis of biolubricant with required constraints and the optimum solution was found at the highest desirability that reached 100%. The optimum conditions to obtain the highest yield of 91.5% were found at a temperature of 141 °C, a reaction time of 3.3 h, and a FAME to Alcohol molar ratio of 2:1. %. Tables 8 and 9 illustrate the conclusion of the optimization constraints and the solution results with their desirability respectively.

**Table 8:** Parameters constraints.

Name	Goal	Lower limit	Upper limit	Lower weight	Upper weight	Importance
A:Temperature	In range	100	150	1	1	3
B:Time	In range	1	4	1	1	4
C:FAME/Alcohol	Minimize	2	4	1	1	3
Yield	Maximize	26.5	89	1	1	5

**Table 9:** Optimization results of biolubricant production.

Number	Temperature	Time	FAME/alcohol	Yield	Desirability	
1	141.044	3.347	2	91.569	1.000	Selected
2	146.919	2.824	2	89.493	1.000	
3	137.260	3.849	2	91.167	1.000	
4	145.117	3.893	2	93.274	1.000	
5	140.453	3.403	2	91.603	1.000	
6	146.961	3.347	2	93.035	1.000	
7	134.82	3.963	2	90.033	1.000	
8	139.183	3.855	2	91.847	1.000	
9	136.652	3.692	2	90.898	1.000	
10	134.431	3.508	2	89.501	1.000	

Five runs were performed at the optimum conditions to assess their validity. The mean biolubricant yield was found to be 91.68% with a standard deviation equal to 1.75%, which proves the validity of the suggested model. The error percentage was found to be 0.122%.

#### Characteristics of optimized biolubricant

##### Fourier transform infrared spectroscopy analysis of biolubricant

**Fig. 10:**
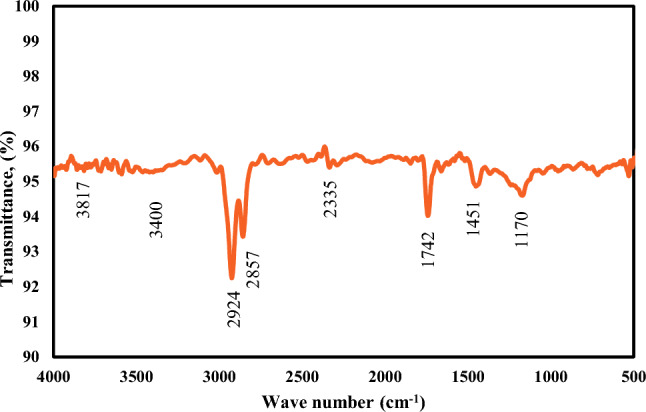
Fourier transform infrared spectroscopy (FTIR) analysis of the produced biolubricant.

Figure 10 shows the FTIR of the produced biolubricant. There are two strong peaks 2924 cm^−1^ and 2857 cm^−1^ that represent the stretching bond of CH_2_ and CH_3_ respectively. The functional group of the produced biolubricant can be identified by the presence of the peak of 1742 cm^−1^ as the ester carbonyl group. While 1451 cm^−1^ confirms the presence of CH alkanes and the band at 1170 cm^−1^ shows the existence of stretching C–O ester. The absorption peaks from 3400 to 3817 cm^−1^ suggest the presence of (O–H) groups from some polyol molecules (ethylene glycol)^36,70,72^.

##### Properties of produced biolubricant

Table 10 shows the characteristics of the produced biolubricant. It can be noted after comparing the previous studies of producing biolubricants from reacting different methyl esters with ethylene glycol that the viscosity was similar to the ISO VG 10 which is compatible with the usage of low-pressure hydraulic oil and spindle bearing with high-speed^73,74^. The high pour point makes the biolubricant not suitable for areas with very low temperatures such as subtropical areas and additives should be added to the biolubricant before usage. The comparison between produced biolubricant and standard specifications.

**Table 10:** Properties of different biolubricants and standard grades of lubricants.

Parameters	Current Study	Soybean^74^	Palm Kernel^74^	Sesame crude oil^75^	ISO VG 10^34^	ISO VG 32^30^
Kinematic viscosity, cSt, @ 40 °C	10.35	27.64	25.315	27.33	> 10	> 28.8
Kinematic viscosity, cSt, @ 100 °C	3.12	4.64	4.315	6.33	> 2.6	> 4.1
Flashpoint, °C	192	-	-	130	> 177	> 204
Viscosity index	183.6	305	215	193	> 74	> 90
Pour point, °C	− 9	− 15	− 9	− 15	− 39	< − 10
Carbon residue, wt.%	1.5	–	–	–	–	–
Ash content, wt.%	0.76					–

##### Reusability of catalyst

**Fig. 11:**
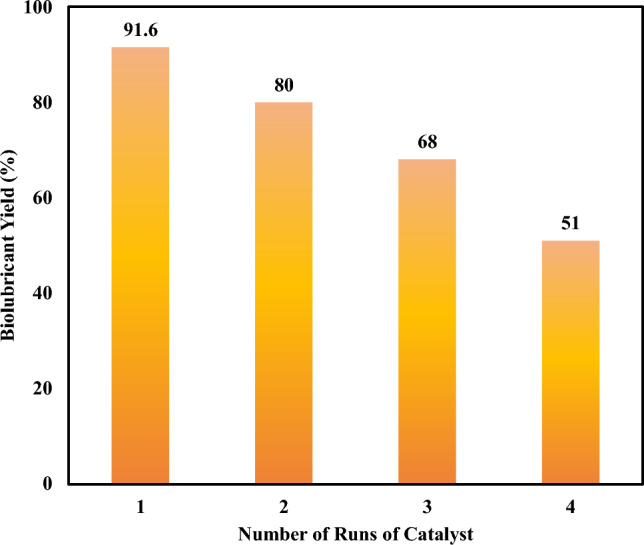
Reusability of the catalyst.

The yield of biolubricant reduced from 91.6% after using it for the first time to 80% after the second run then followed by 68% and 51% respectively as shown in Fig. 11. The reasons for this behavior due to the decreasing the active site of the catalyst such as contamination with the produced methanol that causes the decrease of the activity of the catalyst and also loss of the active sites as a result of leaching of the solid catalyst that converted into a liquid state. According to the obtained results, the catalyst can be used three times only and then replaced with a fresh catalyst.

## Process simulation of biolubricant production

The material and energy balance for the whole process simulated using Aspen Plus for the production of biolubricant from the blending of animal fats and waste cooking oil was illustrated in Tables S1 to S7. The conversion of free fatty acid present in animal fats into biodiesel and water in an esterification reactor is about 46% (to reduce the free fatty acid from 7.5 to 4%). The second conversion reactor (first transesterification reaction) is used to produce biodiesel from the blending of treated animal fat and waste cooking oil in the presence of homogenous NaOH as a catalyst with a conversion of 94% according to the results of experimental work. The produced biodiesel was converted into biolubricant after reacting with ethylene glycol in the presence of calcium oxide as a catalyst with a conversion of 91.6%. Calcium oxide was added as a heterogeneous catalyst in the double transesterification reactor which is removed after the reaction performed using a filter as shown in Fig. 3. The produced methanol from transesterification (II) was separated from the biolubricant using a flash separator to produce methanol vapor at the top stream. The biolubricant product obtained after eliminating the residual catalyst using a filter has a purity of 87 wt.%.

## Economic evaluation of biolubricant production

After finding the optimum condition for biolubricant production, studying the characteristics of the product, and performing process simulation using Aspen Plus, the values of fixed and working capital investments, total manufacturing cost, IRR, and breakeven point are determined. Table 11 shows the cost of raw materials and utilities used for biolubricant production per kg.

**Table 11:** Cost of raw materials and utilities.

Raw material	Cost of $/kg
Ethylene glycol	0.057
Sodium hydroxide	0.005
Animal fats	0.258
Sulfuric acid	5E−05
Waste cooking oil	1.033
Methanol	0.084
Calcium oxide	0.002
Total	1.439
Utilities	Cost of $/kg
Electricity	0.0027
Water	0.00018
Steam	0.00477
Hot oil	0.0319
Total	0.03955

**Fig. 12:**
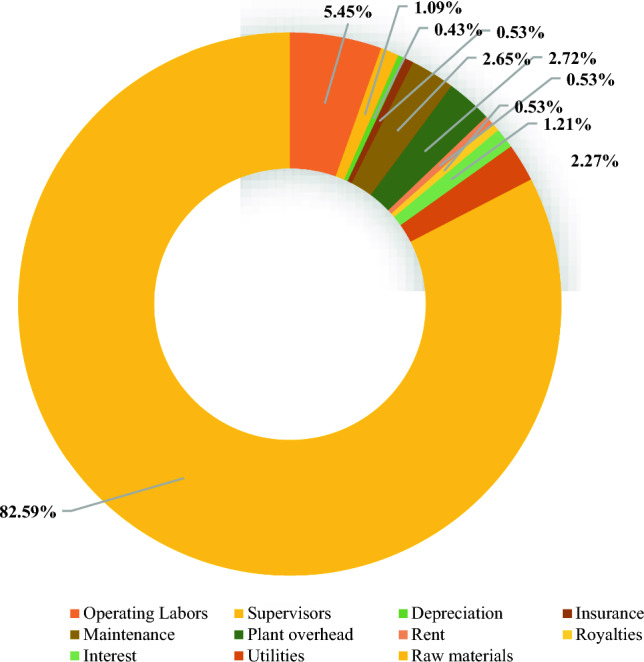
Total production cost of biolubricant.

Table 12 shows the cost of the equipment cost that used in the production of biolubricant including tanks of raw materials, heaters, coolers, reactors, pumps, and separators. Figure 12 illustrates the total production cost (TPC) per kg of biolubricant including the cost of raw materials, utilities, plant overhead, depreciation, insurance, and maintenance. This figure indicated that the raw materials have the highest cost representing about 82.6% of the TPC.

**Table 12:** Cost of purchased equipment.

Equipment	Number of equipment	Equipment cost ($)
Animal fat tank	1	5350
Sulfuric acid tank	1	1667
Methanol tank	1	6000
WCO tank	1	18,233
NaOH tank	1	22,350
Water tank	1	500
Ethylene glycol tank	1	16,233
CaO tank	1	22,350
Glycerol tank	1	10,917
Disposal CaO tank	1	22,350
Biolubricant tank	1	5467
Washing tank	1	189,500
Heaters	3	265,400
Coolers	3	13,590
Reactor 1	1	564,684
Reactor 2	1	30,333
Reactor 3	1	11,450
Mixers	4	476,994
Pumps	4	63,817
Separators	4	135,992
Filter	1	363,218
Total		2,246,395

The economic analysis of the biolubricant product plant was determined according to the financial metrics which are the internal rate of return (IRR), net present value (NPV), and payback period (PBP) as illustrated in Table 13. According to the obtained results, the NPV was found to be $30,343,195 indicating the project is feasibly economical and reliable. The positive value of NPV represents that the project is predicted to produce a profit greater than the total capital investment decreasing loss risk. The internal rate of return was determined to be 67.5%, the higher IRR implies that the project is profitable. The payback period was found to be 1.48 years, revealing the time required to recover the initial.

**Table 13:** Summary of the total production cost of biolubricant production.

Item	Value	Unit
Total physical plant cost (PPC)	7,637,741	($)
Fixed capital investment (FCI)	11,074,725	($)
Working capital investment (WCI)	1,661,208	($)
Total capital investment (TCI)	12,735,934	($)
Direct cost	1.744	($/kg)
Indirect cost	0.349	($/kg)
Total production cost	2.09	($/kg)
Fixed production cost	3,164,896	$/year
Total production per year	11,982,600	kg
Total revenue	27,140,589	($/year)
Selling price of biolubricant	2.4	$/kg
Selling price of glycerol	0.265	$/kg
Profit	11,029,664	$/year
Net profit	8,603,138	$/year
IRR	67.6	%
Profitability	27	%
NPV	30,343,195	$
Payback period	1.48	Year

## Conclusion

Limited energy sources are a major challenge. Future energy scenarios suggest a significant role for biomass energy. Biolubricant was produced by transesterification of fatty acids methyl ester (FAME) and alcohol (ethylene glycol). FAME was produced after blending 20% animal fats and 80% WCO with methanol at a temperature of 60 °C, a reaction time of 90 min, and a ratio of 6:1 of the molar ratio of methanol to oil. Response surface methodology was used to optimize the process parameters of biolubricant production including temperature, time, and molar ratio FAME to alcohol. The highest yield of biolubricant under specific constraints was achieved at a temperature of 141 °C, a reaction time of 3.3 h, and a FAME to alcohol molar ratio of 2:1. The produced biolubricant has a kinematic viscosity at 40 °C 10.35 cSt and viscosity index of 183.6 which is compatible with the standard specification of ISO VG 10. Aspen Plus version 11 was used to simulate the production process of biolubricant which can be applied on the industrial scale with a suitable mass balance. Economic analysis was performed in the production process and the results indicated that the project is feasibly economical and reliable. In the future, improving the viscosity index and pour point of biolubricant using different additives will be studied to be used in heavy equipment.

## Supplementary Information

Supplementary Information.

## Data Availability

The data supporting the findings of this study are available in the manuscript.
