# Supplementary material for: Box-Behnken design (BBD) for optimization and simulation of biolubricant production from biomass using aspen plus with techno-economic analysis
Source: Sci Rep. 2024 Sep 18;14:21769. doi: 10.1038/s41598-024-71266-w (PMC11413246; doi:10.1038/s41598-024-71266-w)
Supplement: Supplementary file 1 — Supplementary Information. [file 41598_2024_71266_MOESM1_ESM.docx]

Table S1: Mass balance results of biolubricant production from aspen plus

| Stream Number | FATS | MEOH | H_2_SO_4_ | 3 | 1 |
| --- | --- | --- | --- | --- | --- |
| Temperature (℃) | 25 | 25 | 25 | 25 | 60 |
| Pressure (kPa) | 101.325 | 101.325 | 101.325 | 101.325 | 91.300 |
| Mass Vapor Fraction | 0.000 | 0.000 | 0.000 | 0.000 | 0.000 |
| Mass Enthalpy (kJ/kg) | -2371.628 | -1080.566 | -8088.930 | -1182.728 | -2308.086 |
| Mass Density (kg/cum) | 755.246 | 645.768 | 1834.061 | 652.010 | 735.328 |
| Enthalpy Flow (kJ/h) | -1185814.1 | -182615.6 | -20222.3 | -202837.9 | -1154043.2 |
| Average MW | 770.338 | 32.042 | 98.079 | 32.360 | 770.338 |
| Mass Flows (kg/h) | 500.0 | 169.0 | 2.5 | 171.5 | 500.0 |
| Mole Flows (kmol/h) | 0.65 | 5.27 | 0.03 | 5.30 | 0.65 |
| Component Mole Fractions |  |  |  |  |  |
| NaOH | 0.000 | 0.000 | 0.000 | 0.000 | 0.000 |
| CaO | 0.000 | 0.000 | 0.000 | 0.000 | 0.000 |
| H_2_SO_4_ | 0.000 | 0.000 | 1.000 | 0.005 | 0.000 |
| CH_3_OH | 0.000 | 1.000 | 0.000 | 0.995 | 0.000 |
| Ethylene Glycol | 0.000 | 0.000 | 0.000 | 0.000 | 0.000 |
| Na_2_SO_4_ | 0.000 | 0.000 | 0.000 | 0.000 | 0.000 |
| Glycerol | 0.000 | 0.000 | 0.000 | 0.000 | 0.000 |
| Water | 0.000 | 0.000 | 0.000 | 0.000 | 0.000 |
| TRIOL-01 | 0.809 | 0.000 | 0.000 | 0.000 | 0.809 |
| Soap (Sodium Oleate) | 0.000 | 0.000 | 0.000 | 0.000 | 0.000 |
| Oleic Acid | 0.191 | 0.000 | 0.000 | 0.000 | 0.191 |
| Biolubricant | 0.000 | 0.000 | 0.000 | 0.000 | 0.000 |
| Methyl Oleate (Biodiesel) | 0.000 | 0.000 | 0.000 | 0.000 | 0.000 |

Table S2: Mass balance results of biolubricant production from aspen plus

| Stream Number | 2 | 4 | 5 | 6 | 7 |
| --- | --- | --- | --- | --- | --- |
| Temperature (℃) | 60 | 60 | 105 | 105 | 105 |
| Pressure (kPa) | 101.325 | 101.325 | 91.300 | 91.300 | 91.300 |
| Mass Vapor Fraction | 0.000 | 0.219 | 0.247 | 1.000 | 0.000 |
| Mass Enthalpy (kJ/kg) | -2308.040 | -1782.091 | -1669.340 | 41.847 | -2229.236 |
| Mass Density (kg/cum) | 735.314 | 5.306 | 3.741 | 0.926 | 635.437 |
| Enthalpy Flow (kJ/h) | -1154020.1 | -1196674.4 | -1120961.8 | 6927.7 | -1127889.4 |
| Average MW | 770.338 | 112.879 | 112.879 | 31.905 | 665.635 |
| Mass Flows (kg/h) | 500.0 | 671.5 | 671.5 | 165.5 | 506.0 |
| Mole Flows (kmol/h) | 0.65 | 5.95 | 5.95 | 5.19 | 0.76 |
| Component Mole Fractions |  |  |  |  |  |
| NaOH | 0.000 | 0.000 | 0.000 | 0.000 | 0.000 |
| CaO | 0.000 | 0.000 | 0.000 | 0.000 | 0.000 |
| H_2_SO_4_ | 0.000 | 0.004 | 0.004 | 0.000 | 0.033 |
| CH_3_OH | 0.000 | 0.877 | 0.877 | 0.990 | 0.105 |
| Ethylene Glycol | 0.000 | 0.000 | 0.000 | 0.000 | 0.000 |
| Na_2_SO_4_ | 0.000 | 0.000 | 0.000 | 0.000 | 0.000 |
| Glycerol | 0.000 | 0.000 | 0.000 | 0.000 | 0.000 |
| Water | 0.000 | 0.010 | 0.010 | 0.010 | 0.007 |
| TRIOL-01 | 0.809 | 0.088 | 0.088 | 0.000 | 0.691 |
| Soap (Sodium Oleate) | 0.000 | 0.000 | 0.000 | 0.000 | 0.000 |
| Oleic Acid | 0.191 | 0.011 | 0.011 | 0.000 | 0.088 |
| Biolubricant | 0.000 | 0.000 | 0.000 | 0.000 | 0.000 |
| Methyl Oleate (Biodiesel) | 0.000 | 0.010 | 0.010 | 0.000 | 0.075 |

Table S3: Mass balance results of biolubricant production from aspen plus

| Stream Number | 8 | WCO | 9 | MEOH1 | NaOH |
| --- | --- | --- | --- | --- | --- |
| Temperature (℃) | 105 | 25 | 42 | 25 | 25 |
| Pressure (kPa) | 111.300 | 125.000 | 111.300 | 111.300 | 101.325 |
| Mass Vapor Fraction | 0.000 | 0.000 | 0.000 | 0.000 | 0.000 |
| Mass Enthalpy (kJ/kg) | -2229.130 | -2330.701 | -2310.194 | -1080.566 | -10645.77 |
| Mass Density (kg/cum) | 635.410 | 909.746 | 825.502 | 645.768 | 2101.611 |
| Enthalpy Flow (kJ/h) | -1127835.6 | -4661402.9 | -5789238.4 | -497060.2 | -399216.3 |
| Average MW | 665.635 | 885.449 | 830.103 | 32.042 | 39.997 |
| Mass Flows (kg/h) | 506.0 | 2000.0 | 2506.0 | 460.0 | 37.5 |
| Mole Flows (kmol/h) | 0.76 | 2.26 | 3.02 | 14.36 | 0.94 |
| Component Mole Fractions |  |  |  |  |  |
| NaOH | 0.000 | 0.000 | 0.000 | 0.000 | 1.000 |
| CaO | 0.000 | 0.000 | 0.000 | 0.000 | 0.000 |
| H_2_SO_4_ | 0.033 | 0.000 | 0.008 | 0.000 | 0.000 |
| CH_3_OH | 0.105 | 0.000 | 0.026 | 1.000 | 0.000 |
| Ethylene Glycol | 0.000 | 0.000 | 0.000 | 0.000 | 0.000 |
| Na_2_SO_4_ | 0.000 | 0.000 | 0.000 | 0.000 | 0.000 |
| Glycerol | 0.000 | 0.000 | 0.000 | 0.000 | 0.000 |
| Water | 0.007 | 0.000 | 0.002 | 0.000 | 0.000 |
| TRIOL-01 | 0.691 | 1.000 | 0.922 | 0.000 | 0.000 |
| Soap (Sodium Oleate) | 0.000 | 0.000 | 0.000 | 0.000 | 0.000 |
| Oleic Acid | 0.088 | 0.000 | 0.022 | 0.000 | 0.000 |
| Biolubricant | 0.000 | 0.000 | 0.000 | 0.000 | 0.000 |
| Methyl Oleate (Biodiesel) | 0.075 | 0.000 | 0.019 | 0.000 | 0.000 |

Table S4: Mass balance results of biolubricant production from aspen plus

| Stream Number | 10 | 11 | 12 | 13 | 15 |
| --- | --- | --- | --- | --- | --- |
| Temperature (℃) | 41 | 60 | 60 | 30 | 60 |
| Pressure (kPa) | 101.325 | 101.300 | 101.325 | 101.325 | 101.325 |
| Mass Vapor Fraction | 0.268 | 0.943 | 0.031 | 0.000 | 0.000 |
| Mass Enthalpy (kJ/kg) | -1275.410 | -544.787 | -2629.285 | -2249.271 | -7657.509 |
| Mass Density (kg/cum) | 4.600 | 1.244 | 35.586 | 755.848 | 1286.024 |
| Enthalpy Flow (kJ/h) | -912661.7 | -389840.4 | -8470335.9 | -6608859.5 | -2169472.4 |
| Average MW | 32.472 | 32.472 | 128.575 | 137.204 | 77.817 |
| Mass Flows (kg/h) | 715.6 | 715.6 | 3221.5 | 2938.2 | 283.3 |
| Mole Flows (kmol/h) | 22.04 | 22.04 | 25.06 | 21.42 | 3.64 |
| Component Mole Fractions |  |  |  |  |  |
| NaOH | 0.043 | 0.043 | 0.033 | 0.000 | 0.225 |
| CaO | 0.000 | 0.000 | 0.000 | 0.000 | 0.000 |
| H_2_SO_4_ | 0.000 | 0.000 | 0.000 | 0.000 | 0.000 |
| CH_3_OH | 0.955 | 0.955 | 0.530 | 0.620 | 0.000 |
| Ethylene Glycol | 0.002 | 0.002 | 0.002 | 0.000 | 0.015 |
| Na_2_SO_4_ | 0.000 | 0.000 | 0.001 | 0.000 | 0.007 |
| Glycerol | 0.000 | 0.000 | 0.104 | 0.000 | 0.719 |
| Water | 0.000 | 0.000 | 0.005 | 0.000 | 0.034 |
| TRIOL-01 | 0.000 | 0.000 | 0.007 | 0.008 | 0.000 |
| Soap (Sodium Oleate) | 0.000 | 0.000 | 0.003 | 0.003 | 0.000 |
| Oleic Acid | 0.000 | 0.000 | 0.000 | 0.000 | 0.000 |
| Biolubricant | 0.000 | 0.000 | 0.000 | 0.000 | 0.000 |
| Methyl Oleate (Biodiesel) | 0.000 | 0.000 | 0.316 | 0.369 | 0.000 |

Table S5: Mass balance results of biolubricant production from aspen plus

| Stream Number | H_2_O | WW | BIODIES | 16 | EG |
| --- | --- | --- | --- | --- | --- |
| Temperature (℃) | 80 | 64 | 64 | 141 | 25 |
| Pressure (kPa) | 101.325 | 101.325 | 101.325 | 101.325 | 101.325 |
| Mass Vapor Fraction | 0.000 | 0.000 | 0.000 | 0.000 | 0.000 |
| Mass Enthalpy (kJ/kg) | -15630.712 | -15595.515 | -2378.134 | -2203.490 | -7386.163 |
| Mass Density (kg/cum) | 939.182 | 951.387 | 781.482 | 730.242 | 1119.128 |
| Enthalpy Flow (kJ/h) | -39264349.4 | -39493605.6 | -5927625.3 | -5492317.9 | -1849273.5 |
| Average MW | 18.015 | 18.153 | 308.676 | 308.676 | 62.068 |
| Mass Flows (kg/h) | 2512.0 | 2532.4 | 2492.6 | 2492.6 | 250.4 |
| Mole Flows (kmol/h) | 139.44 | 139.50 | 8.07 | 8.07 | 4.03 |
| Component Mole Fractions |  |  |  |  |  |
| NaOH | 0.000 | 0.000 | 0.000 | 0.000 | 0.000 |
| CaO | 0.000 | 0.000 | 0.000 | 0.000 | 0.000 |
| H_2_SO_4_ | 0.000 | 0.000 | 0.000 | 0.000 | 0.000 |
| CH_3_OH | 0.000 | 0.000 | 0.000 | 0.000 | 0.000 |
| Ethylene Glycol | 0.000 | 0.000 | 0.000 | 0.000 | 1.000 |
| Na_2_SO_4_ | 0.000 | 0.000 | 0.000 | 0.000 | 0.000 |
| Glycerol | 0.000 | 0.000 | 0.000 | 0.000 | 0.000 |
| Water | 1.000 | 1.000 | 0.000 | 0.000 | 0.000 |
| TRIOL-01 | 0.000 | 0.000 | 0.021 | 0.021 | 0.000 |
| Soap (Sodium Oleate) | 0.000 | 0.000 | 0.000 | 0.000 | 0.000 |
| Oleic Acid | 0.000 | 0.000 | 0.000 | 0.000 | 0.000 |
| Biolubricant | 0.000 | 0.000 | 0.000 | 0.000 | 0.000 |
| Methyl Oleate (Biodiesel) | 0.000 | 0.000 | 0.979 | 0.979 | 0.000 |

Table S6: Mass balance results of biolubricant production from aspen plus

| Stream Number | 17 | CAO | 18 | 22 | 19 |
| --- | --- | --- | --- | --- | --- |
| Temperature (℃) | 130 | 25 | 141 | 141 | 141 |
| Pressure (kPa) | 101.325 | 101.325 | 101.325 | 101.325 | 101.325 |
| Mass Vapor Fraction | 0.000 | 0.000 | 0.079 | 0.000 | 1.000 |
| Mass Enthalpy (kJ/kg) | -2676.557 | -11325.240 | -2382.338 | -2591.694 | 71.847 |
| Mass Density (kg/cum) | 731.420 | 3297.632 | 11.768 | 387.098 | 0.952 |
| Enthalpy Flow (kJ/h) | -7341591.4 | -357198.1 | -6609941.8 | -6625610.3 | 15668.5 |
| Average MW | 226.524 | 56.077 | 227.250 | 467.702 | 32.341 |
| Mass Flows (kg/h) | 2742.9 | 31.5 | 2774.6 | 2556.5 | 218.1 |
| Mole Flows (kmol/h) | 12.11 | 0.56 | 12.21 | 5.47 | 6.74 |
| Component Mole Fractions |  |  |  |  |  |
| NaOH | 0.000 | 0.000 | 0.000 | 0.000 | 0.000 |
| CaO | 0.000 | 1.000 | 0.046 | 0.103 | 0.000 |
| H_2_SO_4_ | 0.000 | 0.000 | 0.000 | 0.000 | 0.000 |
| CH_3_OH | 0.000 | 0.000 | 0.567 | 0.044 | 0.992 |
| Ethylene Glycol | 0.333 | 0.000 | 0.028 | 0.052 | 0.008 |
| Na_2_SO_4_ | 0.000 | 0.000 | 0.000 | 0.000 | 0.000 |
| Glycerol | 0.000 | 0.000 | 0.000 | 0.000 | 0.000 |
| Water | 0.000 | 0.000 | 0.000 | 0.000 | 0.000 |
| TRIOL-01 | 0.014 | 0.000 | 0.014 | 0.031 | 0.000 |
| Soap (Sodium Oleate) | 0.000 | 0.000 | 0.000 | 0.000 | 0.000 |
| Oleic Acid | 0.000 | 0.000 | 0.000 | 0.000 | 0.000 |
| Biolubricant | 0.000 | 0.000 | 0.303 | 0.676 | 0.000 |
| Methyl Oleate (Biodiesel) | 0.653 | 0.000 | 0.042 | 0.095 | 0.000 |

Table S7: Mass balance results of biolubricant production from aspen plus

| Stream Number | RESI-CAO | BIOLUB | MEOH2 | 20 | 21 |
| --- | --- | --- | --- | --- | --- |
| Temperature (℃) | 141 | 141 | 30 | 60 | 60 |
| Pressure (kPa) | 101.325 | 101.325 | 95.000 | 101.325 | 101.325 |
| Mass Vapor Fraction | 0.000 | 0.000 | 0.000 | 0.978 | 1.000 |
| Mass Enthalpy (kJ/kg) | -11215.523 | -2484.756 | -1062.726 | -75.132 | -26.098 |
| Mass Density (kg/cum) | 3277.512 | 382.911 | 640.196 | 1.200 | 1.167 |
| Enthalpy Flow (kJ/h) | -351188.9 | -6274421.4 | -451978.0 | -16385.1 | -4320.4 |
| Average MW | 56.118 | 514.494 | 32.042 | 32.341 | 31.905 |
| Mass Flows (kg/h) | 31.3 | 2525.2 | 425.3 | 218.1 | 165.5 |
| Mole Flows (kmol/h) | 0.56 | 4.91 | 13.27 | 6.74 | 5.19 |
| Component Mole Fractions |  |  |  |  |  |
| NaOH | 0.000 | 0.000 | 0.000 | 0.000 | 0.000 |
| CaO | 1.000 | 0.001 | 0.000 | 0.000 | 0.000 |
| H_2_SO_4_ | 0.000 | 0.000 | 0.000 | 0.000 | 0.000 |
| CH_3_OH | 0.000 | 0.049 | 1.000 | 0.992 | 0.990 |
| Ethylene Glycol | 0.000 | 0.058 | 0.000 | 0.008 | 0.000 |
| Na_2_SO_4_ | 0.000 | 0.000 | 0.000 | 0.000 | 0.000 |
| Glycerol | 0.000 | 0.000 | 0.000 | 0.000 | 0.000 |
| Water | 0.000 | 0.000 | 0.000 | 0.000 | 0.010 |
| TRIOL-01 | 0.000 | 0.034 | 0.000 | 0.000 | 0.000 |
| Soap (Sodium Oleate) | 0.000 | 0.000 | 0.000 | 0.000 | 0.000 |
| Oleic Acid | 0.000 | 0.000 | 0.000 | 0.000 | 0.000 |
| Biolubricant | 0.000 | 0.753 | 0.000 | 0.000 | 0.000 |
| Methyl Oleate (Biodiesel) | 0.000 | 0.105 | 0.000 | 0.000 | 0.000 |
